# The Association Between Perceived Discrimination and Body Mass Index (BMI) Among Asian American Women Before and During the COVID-19 Pandemic

**DOI:** 10.3390/ijerph22050661

**Published:** 2025-04-22

**Authors:** Uyen-Sa D. T. Nguyen, Hyeouk Chris Hahm, Sarpong Boateng, Phuong Khanh Tran, Rajesh Gururaghavendran, Yvette C. Cozier

**Affiliations:** 1 Department of Population & Community Health, College of Public Health, University of North Texas Health Science Center, Fort Worth, TX 76107, USA; uyen-sa.nguyen@unthsc.edu (U.-S.D.T.N.); sarpongboateng@my.unthsc.edu (S.B.); 2 School of Social Work, Boston University, Boston, MA 02115, USA; hahm@bu.edu; 3 Yale New Haven Health, Bridgeport Hospital, Bridgeport, CT 06610, USA; 4 Department of Electrical and Computer Engineering, College of Engineering, Boston University, Boston, MA 02215, USA; kontact@bu.edu; 5 Department of Health Outcomes and Biomedical Informatics, Institute for Child Health Policy, College of Medicine, University of Florida, Gainesville, FL 32611, USA; rajeshgururaghav@ufl.edu; 6 School of Public Health, Boston University, Boston, MA 02215, USA

**Keywords:** Asian, Asian American, obesity, body mass index, racism, discrimination

## Abstract

We examined the association between perceived discrimination and body mass index (BMI) in Asian American (AA) women before and during the COVID-19 pandemic. The data used were from the Epidemiology/Epigenetics of Asian Women’s Action for Resilience and Empowerment (Epi AWARE) study, which enrolled 157 AA women aged 18 to 59 between December 2019 and September 2022. Two sets of questions measured “everyday” (e.g., “How often do people act as if you are dishonest?”) and “lifetime” (e.g., “unfair treatment due to your race at school”) discrimination. Responses were combined, creating a cumulative score, and dichotomized into “no” versus “any” discrimination. Self-reported height and weight were used to calculate BMI, dichotomized as <25 kg/m^2^ and ≥25 kg/m^2^. Multivariable binomial regression estimated risk ratios (RRs) and 95% confidence intervals (95% CIs). Overall, women reporting any versus no discrimination were more likely to be classified as having a BMI ≥ 25 kg/m^2^ (RR = 2.09; 95% CI: 1.06–4.13). The RR for women who responded during the COVID-19 pandemic (20 March 2020 or later) was 2.65 (95% CI: 0.82–8.58); the RR for pre-pandemic response was 1.93 (95% CI: 0.75–5.01). Our data suggest that experiences of racism may impact BMI among AA women. Further research is needed to identify mechanisms and design appropriate interventions.

## 1. Introduction

Obesity is a major public health concern [1,2,3]. Body mass index (BMI) serves as a practical measure of body fat in both clinical and epidemiological settings [4]. Higher BMI is associated with adverse health outcomes, including type-2 diabetes (T2 DM), cardiovascular disease (CVD), and mortality [5,6,7]. BMI is calculated using height and weight (kg/m^2^) [8]. The World Health Organization (WHO) defines a BMI between 25 kg/m^2^ and 29.9 kg/m^2^ as overweight and a BMI of ≥30 kg/m^2^ as obese [9].

From 2017 to 2018, Asian Americans (AAs) had the lowest obesity prevalence (17.4%) compared with other racial/ethnic groups [10], with BMI values approximately 2–3 kg/m^2^ lower than in Whites [11]. Asians, however, exhibit a heightened susceptibility to developing T2DM and high blood pressure compared to Whites [12,13]. Body composition variations within the population may contribute to this phenomenon [14]. In 2002, WHO recommended revised BMI cut-points for obesity (≥27.5 kg/m^2^) and overweight (≥23 kg/m^2^ and <27.5 kg/m^2^) for Asians to facilitate public health interventions [15]. Thus, identifying risk factors for overweight and obesity is critical for Asian Americans.

Multiple factors contribute to high BMI. These include older age [16], poverty [17], and low physical activity [18,19]. Perceived discrimination has also shown a robust relationship with weight gain and obesity among African Americans [20,21,22,23] and Hispanics [24,25].

Research into the impact of discrimination on AAs remains limited, especially for AA women. One study found a stronger effect of discrimination on CVD risk in AAs than in Blacks, using combined data from the National Survey of American Life (NSAL) and the National Latino and Asian American Study (NLAAS) [26]. Another study found a strong relationship between perceived discrimination and higher BMI among AA men and women in the NLAAS [27]. Data from the NSAL and NLAAS were collected approximately two decades ago and utilized the Everyday Discrimination Scale [28,29]. Neither study, however, assessed lifetime experiences of discrimination, including in employment or housing [28,29,30].

Given that Asians are the fastest-growing racial/ethnic group in the U.S. [31], with increasing rates of anti-Asian discrimination across different social contexts [32,33], there remains a significant gap in our understanding of the impact of discrimination on AA health. A recent study found that 68% of young AA women reported that they or their family members experienced COVID-19-related microaggressions, and 15.2% reported verbal or physical assaults [33]. Lee et al. found increased reports of racial discrimination, hyper-vigilance, and psychosocial stress among AA women during the COVID-19 lockdown [34]. These findings highlight the importance of assessing the impact of discrimination on AA women, overall and during heightened periods of social/cultural unrest. Therefore, our goal was to determine the association of perceived everyday and lifetime discrimination with overweight/obesity in a sample of AA women. We also explored this relationship with respect to COVID-19 pandemic status.

## 2. Materials and Methods

### 2.1. Study Sample

Participants of this study were from the Epidemiology and Epigenetics of Asian Women’s Action for Resilience and Empowerment (Epi AWARE) study. Between December 2019 and September 2022, we enrolled women aged 18 to 59 who identified as Asian American and willing to answer an online questionnaire [34]. Using online questionnaires, we obtained information on demographics, lifestyle factors, childhood and current experiences, and medical conditions. Of the 179 respondents who completed the online questionnaire, we excluded women with missing values for the discrimination questions, weight, or height, leaving 157 women for the current analysis. Participants indicated their informed consent by completing the questionnaires. The study protocol was approved by the Institutional Review Board.

### 2.2. Measures of Perceived Discrimination

We used three distinctive definitions of perceived discrimination: everyday, lifetime, and cumulative discrimination, which combined the everyday and lifetime discrimination measures. The everyday and lifetime discrimination questionnaires were adapted from an original instrument developed by Williams et al. [28,29]. Five items measured the frequency of everyday experiences: *“act as if they are better than you,” “poorer service than other people in restaurants or stores,” “they think you are dishonest,” “they think you are not intelligent,”* and *“they are afraid of you.”* Responses ranged from “never” to “almost every day.” We further dichotomized each question as either never/a few times a year (0) or once a month/once a week/almost every day (1). Respondents were also asked six items measuring lifetime racial discrimination, i.e., any experience of unfair treatment due to race *by the police, on the job, in housing, in the courts, in school,* or *while getting medical care*. Response categories were coded as “yes” (1) or “no” (0). Finally, we created a cumulative discrimination score by summing the dichotomized everyday and lifetime responses and generated tertiles of the combined self-reported perceived discrimination score: tertiles 1 (no), 2 (low), and 3 (high). We modeled the cumulative discrimination score as a dichotomous variable by combining the 2nd and 3rd tertiles into one category indicating any reported discrimination (score ≥ 1) versus tertile 1 (no reported discrimination, score = 0).

### 2.3. Body Mass Index

We calculated BMI using self-reported weight in kilograms (kg) divided by height in square meters (m^2^). BMI was dichotomized into <25 kg/m^2^ (“normal”) and ≥25 kg/m^2^ (“overweight/obese”) [9]. To consider the specific health risks and body composition patterns among Asian populations, we also employed the cut-point of 23 kg/m^2^ [35].

We assessed the validity of self-reported weight and height in a sample of Epi AWARE participants. Between May 2021 and December 2022, 34 participants came to the General Clinical Research Unit at the Boston University School of Medicine for anthropometry and phlebotomy performed by trained clinical staff. There were no significant differences between self-reported and technician-measured weight (pounds): the respective mean (SD) values were 128.4 (4.43) versus 130.5 (3.81) (*p =* 0.72). Similarly, there were no significant differences according to self-reported and technician-measured height (inches): 63.3 (0.31) vs. 63.1 (0.32), respectively (*p =* 0.67).

### 2.4. Covariates

Covariates included current age (years), completed education (<12, ≥12 years), annual household income (<USD 15,000, USD 15,000–25,000, USD 25,001–35,000, USD 35,001–50,000, USD 50,001–100,000, ≥USD 10,000), marital status (married, unmarried), sexual orientation (heterosexual, not heterosexual), country of birth (U.S., non-U.S.), alcohol intake (ever or missing data versus never), smoking (ever versus never), moderate physical activity (0, <1, 1–2, 3–4, ≥5 h/week), sedentary activity (0, <1, 1–2, 3–4, ≥5 h/day), and history of poverty (yes if participant had ever received public assistance or welfare and/or lacked money for food or housing as a child, teenager, or adult; else no). Questionnaire completion date was classified as before 20 March 2020 (pre-COVID-19 pandemic) and on or after that date (during-COVID-19 pandemic).

### 2.5. Statistical Analysis

Descriptive statistics for ordinal and categorical variables were summarized using frequencies and proportions, while continuous variables were summarized using means and standard deviations (SDs). We examined participant characteristics across tertiles of the cumulative discrimination score. We then used binomial regression to estimate risk ratios (RRs) and Wald-statistic 95% confidence intervals (CIs). First, we examined the unadjusted RRs for the association between cumulative perceived discrimination and high BMI (≥25 kg/m^2^) and then examined them separately for each item indicating everyday or lifetime racism and high BMI. In building our models for each outcome, we added each covariate, retaining those variables which changed the RR estimate by ≥10%. We also conducted stepwise multivariable binomial regression to confirm our model building. Our final adjusted model included age (1-year intervals), history of poverty, and alcohol consumption (including the missing alcohol data indicator). We also explored the impact of the COVID-19 pandemic on the association between cumulative discrimination and BMI by stratifying on the questionnaire completion date. All analyses were repeated using the BMI cut-point of 23 kg/m^2^ [27]. All statistical tests were two-sided, and a significance level of 0.05 was selected prior to our analyses. All analyses were performed using SAS version 9.3 (SAS Institute, Cary, NC, USA).

## 3. Results

### 3.1. Participant Characteristics

The average BMI was 23.1 (SD = 3.96), with 22.9% of participants having a BMI ≥ 25 kg/m^2^ and 40.1% having a BMI ≥ 23 kg/m^2^. Overall, the mean age was 29.1 years (SD = 9.34). Table 1 summarizes the sample characteristics by tertiles of cumulative perceived discrimination. Participants in the highest tertile of perceived discrimination were more likely to be unmarried, engage in sedentary activities, have a history of poverty, be U.S.-born, have never smoked cigarettes, and have a history of alcohol consumption. Sample characteristics by a binary classification of the perceived discrimination score are presented in Supplementary Table S1.

### 3.2. Cumulative Discrimination and the Risk of Overweight/Obesity

**Figure 1:**
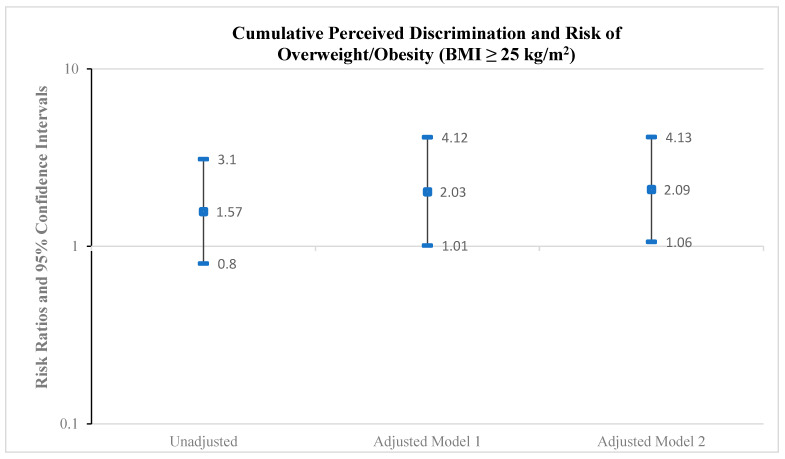
Risk ratios and 95% confidence intervals for the association between cumulative perceived discrimination (any discrimination versus no discrimination) and risk of overweight/obesity (BMI ≥ 25 kg/m^2^), Epi AWARE Study (n = 157); Model 1: adjusted for age and poverty; Model 2: adjusted for age, poverty, and alcohol.

**Figure 2:**
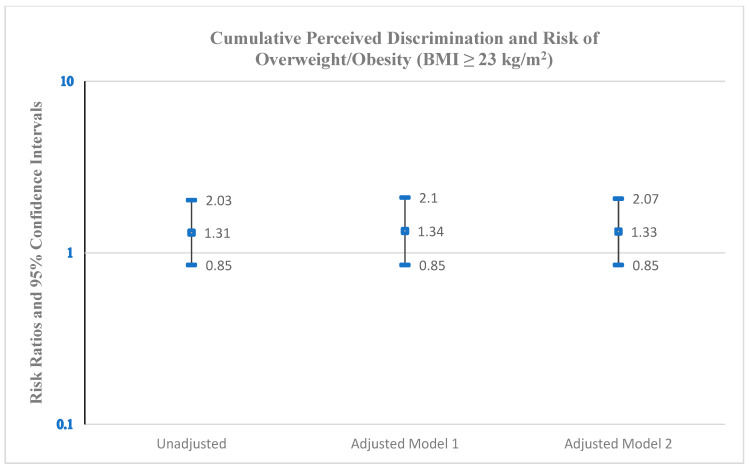
Risk ratios and 95% confidence intervals for the association between cumulative perceived discrimination (any discrimination versus no discrimination) and risk of overweight/obesity (BMI ≥ 23 kg/m^2^), Epi AWARE Study (n = 157); Model 1: adjusted for age and history of poverty; Model 2: adjusted for age, history of poverty, and alcohol consumption.

As shown in Figure 1, the cumulative discrimination score was positively associated with the risk of overweight/obesity. Based on a BMI cut-point of 25 kg/m^2^, the RR was 2.03 (95% CI: 1.01–4.12) for any discrimination compared with no discrimination on overweight/obesity, adjusting for age and history of poverty (Model 1). Further adjustment for alcohol consumption (Model 2) minimally increased the RR to 2.09 (95% CI: 1.06–4.13). The corresponding RR measures for the BMI cut-point of 23 kg/m^2^ (Figure 2) R were 1.34 (95% CI: 0.85–2.10) (Model 1) and 1.33 (95% CI: 0.85–2.07) (Model 2).

### 3.3. Everyday Discrimination and the Risk of Overweight/Obesity

We explored the individual everyday discrimination questions and their respective associations with overweight/obesity. All questions were positively associated with a BMI ≥ 25 kg/m^2^ (Table 2). For example, the multivariable-adjusted RR (Model 2) was 3.87 (95% CI: 1.98–7.54) for women reporting people acting as if they were dishonest at least monthly versus never/a few times per year. Similarly, the RR for women reporting people acting afraid of them was 4.73 (95% CI: 1.86–12.05). When we explored these associations using the BMI cut-point of 23 kg/m^2^ (Supplementary Table S2), the RRs were attenuated, with only “.. better than you”, “.. dishonest”, and “.. afraid” remaining statistically significant: 1.53 (95% CI: 1.05–2.25), 2.11 (95% CI: 1.31–3.43), and 2.24 (95% CI: 1.16–4.34), respectively.

### 3.4. Lifetime Discrimination and the Risk of Overweight/Obesity

Table 3 presents the estimated RR of overweight/obesity (BMI cut-point of 25 kg/m^2^) associated with individual lifetime discrimination questions. Positive associations were found for unfair treatment due to race on the job (RR = 1.82, 95% CI: 1.04–3.19) and while receiving medical care (RR = 3.01, 95% CI: 1.63–5.56). When the associations were explored using a BMI cut-point of 23 kg/m^2^ (Supplementary Table S3), only unfair treatment while receiving medical care remained statistically significant (RR = 2.12, 95% CI: 1.46–3.08).

### 3.5. Impact of the COVID-19 Pandemic on the Association Between Racism and BMI

Finally, we examined the association between cumulative perceived discrimination and overweight/obesity by the survey completion date (before 20 March 2020 [n = 78; mean age (SD) = 27.4 (8.6) years] versus on or after 20 March 2020 [n = 79; mean age (SD) = 30.4 (9.9) years], *p =* 0.045) (Table 4). Overall, participants reporting any discrimination had an increased risk of being overweight/obese versus those reporting no discrimination. Although not statistically significant, the magnitude of the association was notably higher for women who responded during the COVID-19 pandemic than for those who responded prior to the pandemic. For example, when we applied a BMI cut-point of 25 kg/m^2^, the multivariable-adjusted RR for women who completed the questionnaire prior to the pandemic was 1.93 (95% CI: 0.75–5.01), while it was 2.65 (95% CI: 0.82–8.58) for women who completed the questionnaire during the pandemic. Using a BMI cut-point of 23 kg/m^2^, the corresponding multivariable-adjusted RRs were 1.06 (95% CI: 0.55–1.83) for questionnaire completion before 20 March 2020 and 1.92 (95% CI: 0.94–3.90) for completion on or after 20 March 2020.

## 4. Discussion

To our knowledge, this is the first study to examine the association between perceived discrimination and overweight/obesity among AA women overall and in relation to the timing of the COVID-19 pandemic. Our results suggest that AA women who reported any perceived discrimination were more likely to be overweight or obese compared to those who reported no discrimination. Additionally, we found that the association was strongest among those women who completed the questionnaire during the pandemic, likely reflecting the pandemic-related increase in hostilities directed against AA, although this relationship was not statistically significant.

We also explored the individual discrimination questions, using both 23 kg/m^2^ and 25 kg/m^2^ cut-points for overweight/obesity. Using 25 kg/m^2^ as the cut-point, the RR for experiencing everyday discrimination (e.g., receiving poorer service than others) monthly or more frequently ranged from 2.72 to 4.73 compared to those who reported such experiences never or only a few times per year. When using the BMI cut-point of 23 kg/m^2^, we found statistically significant associations between three out of five discrimination items (better than you, dishonest, and afraid of you) and overweight/obesity, although the estimates were attenuated. Similarly, the RRs for women who reported racial discrimination related to employment or receiving medical care were 1.82 and 3.01 compared to women who did not, respectively. The association with receiving medical care remained significant when using 23 kg/m^2^ as the cut-point for overweight/obesity.

Our results are consistent with previous research on racial discrimination and increased BMI. In a cross-sectional analysis of the NLAAS, Gee et al. found that among AA men and women (mean (SD) age: 41.1 (15.6) years, 52% women), those who experienced racial discrimination, measured using the Williams Everyday Discrimination Scale [28,29], had 16% greater odds of being overweight and 112% greater odds of being obese compared with those who reported no perceived discrimination [27]. In another cross-sectional analysis of African American male respondents to the NSAL study, Thorpe and colleagues found that those who reported any lifetime experience of racial discrimination (e.g., mistreated/abused by police and being denied a loan) had an obesity prevalence ratio that was 35% higher than those reporting no major experiences [21]. In the same study, men who experienced everyday discrimination (e.g., being perceived as dishonest) also had a 27% higher obesity prevalence ratio than those who did not, but the association was not statistically significant. Longitudinally, a follow-up of Black Brazilian higher education and research civil servants found that those reporting racial discrimination had a 64% higher 4-year risk of obesity compared to those not reporting discrimination [36].

The observed associations between racial discrimination and increased BMI may be attributed to several interconnected stress-related mechanisms. In humans, the hypothalamic–pituitary–adrenal (HPA) axis is the major stress response pathway leading to cortisol secretion [37], and exposure to discrimination can lead to dysregulated HPA function and increased cortisol secretion [25]. Stress-induced cortisol release can increase appetite, insulin resistance, and abdominal fat accumulation [38], and it is positively associated with metabolic syndrome, T2DM, and CVD [39]. Furthermore, individuals experiencing chronic stress may resort to emotional eating as a coping mechanism [40], which has been linked to obesity and weight gain [41]. Both animal and human studies have shown that stress increases the consumption of high-fat, energy-dense “comfort food” [25,42,43,44]. Molina et al. found that discrimination may promote maladaptive behavioral responses, such as poor dietary choices and increased food consumption, which help to relieve stress in the short-term but increase the risk of obesity [25]. In a study of U.S. Black women, those reporting high everyday racism scores were also more likely to report frequent fast-food consumption [20]. Additionally, in a study of the impact of self-report discrimination on brain activity in response to food images, Zhang et al. found that discrimination was associated with increased food-cue reactivity in the regions of the brain involved in reward, motivation, and execution control and led to a preference for unhealthy foods [45]. Thus, discrimination as a stressor may contribute to dysregulation in the brain–gut-microbiome system from food-cue responses, leading to unhealthy eating behaviors that may increase the risk for obesity [45].

Hyper-vigilance or hyper-anticipation of discrimination because of one’s identity is another source of chronic stress [46,47]. Hyper-vigilance is associated with fear and anxiety [48] and can negatively impact health [49,50]. Sawyer et al. found that hyper-vigilance can elicit psychosocial and physiologic stress responses [51]. A qualitative study by Hahm et al. found that pandemic-related anti-Asian racism led both Asians and non-Asians to avoid routine activities outside the home (e.g., grocery shopping) for fear of discrimination [33]. In a study by Lee and colleagues, AA women were reported to experience emotional stress during the COVID-19 pandemic, including feelings of fear, anxiety, and paranoia [34]. This stress resulted from both discriminatory experiences and fear of victimization. In addition, physical inactivity and unhealthy eating habits and lifestyle behaviors, as well as high levels of stress, anxiety, and depression, increased during the COVID-19 pandemic, which has been linked to obesity and weight gain [52]. Studies suggest that these changes in weight and body composition may have persisted beyond the immediate period of the pandemic, potentially having long-term effects on metabolic health and overall well-being [53,54,55]. For Asian American women, increased racism during the COVID-19 pandemic may have exacerbated these issues, potentially contributing to longer-term changes in BMI and related health problems. Given the emerging evidence of the ongoing effects of the pandemic on weight and metabolic health, further research is needed to understand these effects in the context of discrimination and social stressors.

Finally, racial and ethnic minorities may face additional challenges, such as limited access to healthy food choices and safe spaces for physical activity, access to care, and underlying medical conditions, which can also contribute to obesity disparities [56,57,58]. Thus, understanding the impact of factors associated with obesity in AA, particularly AA women, may be important for preventing obesity-related conditions (e.g., T2DM) in this population.

### Limitations and Strengths

Our study has several limitations. First, given the cross-sectional study design, the temporal association between self-reported perceived racial discrimination and high BMI is unclear. Thus, we cannot rule out that being overweight/obese leads to perceived discrimination. Second, our sample size was small. Nevertheless, we observed robust, statistically significant estimates. Moreover, experiences of racial discrimination were ascertained using the Williams instrument [28], which has been shown to have high reliability and validity in Black/African American populations [59]. While results from Chan et al. examining the reliability and validity of the nine-item Everyday Discrimination Scale in the NLAAS indicated very good overall internal consistency for Vietnamese Americans (Cronbach’s α = 0.924) and Chinese Americans (Cronbach’s α = 0.886) [60], AAs report frequently being mistaken as foreign-born [34]. Thus, it is possible that some items included in the everyday and lifetime racism instruments may not have resonated with our AA participants. In addition, the findings may not be generalizable to Asian American women younger than 18 or to middle-aged or older adults.

Our study was also limited by several other factors that we were unable to account for, such as dietary habits, underlying medical conditions, medication history, and access to care. Also, BMI was calculated from self-reported height and weight. In a subsample, we were able to compare self-reported weight and height values with those objectively measured by professionals to ensure accuracy and reduce potential biases in the data. Although the self-reported values did not differ significantly from the technician-measured values, some degree of measurement error is still possible [61]. If present, such non-differential misclassification would likely have biased results toward the null, leading to underestimation of the true association between racial discrimination and overweight/obesity. Social desirability bias may also have influenced how participants reported their height and weight, potentially leading to underestimation of BMI. However, we do not believe that the biased reporting of BMI would have been related to how participants reported their level of self-perceived racism (e.g., that those who reported a lower than actual BMI because of social desirability would have been somehow reporting higher racism). Thus, any misreporting of BMI is likely to have been unrelated to reports of discrimination and would therefore have biased the association toward the null rather than inflated it.

Despite these limitations, a major strength of our study is that we were able to analyze the data as pre-COVID-19 (before 20 March 2020) or during-COVID-19 (on or after 20 March 2020). Our results suggested that the magnitude of the association between perceived discrimination and BMI was stronger during the COVID-19 pandemic, possibly due to the marked increase in anti-Asian discrimination. Finally, we explored the association using two BMI cut-points for obesity/overweight: 25 kg/m^2^ and 23 kg/m^2^. This approach allowed us to compare our findings with previous studies using the traditional cut-point (25 kg/m^2^) while also considering the specific health risks and body composition patterns relevant to Asian populations (23 kg/m^2^).

## 5. Conclusions

Results from this study show that racial discrimination is strongly associated with increased BMI in young AA women. Our results add to the existing evidence that experiences of racism may be associated with the high burden of obesity observed in ethnic minorities in this country, particularly during the COVID-19 pandemic [62]. Further research is needed to examine possible mechanisms to inform targeted interventions and reduce adverse health outcomes in this vulnerable and understudied group.

## Figures and Tables

**Table 1:** Participant characteristics according to cumulative perceived discrimination score in tertiles (none, low, high), Epi AWARE Study (n = 157).

		Cumulative Perceived Discrimination Score		
	Total (n = 157)	Tertile 1 (None) (n = 54)	Tertile 2 (Low) (n = 43)	Tertile 3 (High) (n = 60)
Age (years)				
Mean (SD), range	29.1 (9.4) 18.0, 59.0	30.3 (10.3) 19.0, 59.0	28.4 (9.7) 18.0, 57.0	28.1 (8.2) 18.0, 56.0
	n (%)			
Education (years)				
>12	152 (96.8%)	53 (98.2%)	40 (93.0%)	59 (98.3%)
Sexual Orientation				
Heterosexual	123 (78.3%)	45 (83.3%)	32 (74.4%)	46 (76.7%)
Marital Status				
Unmarried	122 (77.8%)	38 (70.4%)	33 (76.7%)	51 (85.0%)
Moderate Physical Activity/Week				
≥3 h	30 (19.1%)	10 (18.5%)	11 (25.6%)	9 (15.0%)
Sedentary Activity/Day				
≥5 h	100 (63.7%)	34 (63.0%)	21 (48.8%)	45 (75.0%)
Country of Birth				
United States	98 (62.4%)	33 (61.1%)	25 (58.1%)	40 (66.7%)
History of Poverty				
History of poverty	60 (38.2%)	14 (25.9%)	17 (39.5%)	29 (48.3%)
Cigarette Smoking				
Ever smoker	16 (10.2%)	7 (13.0%)	3 (7.0%)	6 (10.0%)
Alcohol Consumption				
Ever drinker	24 (15.3%)	8 (14.8%)	6 (13.9%)	10 (16.7%)
Missing	103 (65.6%)	34 (63.0%)	29 (67.4%)	40 (66.7%)

**Table 2:** Risk ratios and 95% confidence intervals for the association between individual measures of perceived everyday discrimination and overweight/obesity (BMI ≥ 25 kg/m^2^), Epi AWARE Study (n = 157).

			Multivariable-Adjusted Risk Ratios and 95% Confidence Intervals					
	N		Model 1 ^1^			Model 2 ^2^		
	Total	BMI: ≥25 kg/m^2^	RR	95% CI		RR	95% CI	
*In your day-to-day life, how often have any of the following things happened to you?*								
Receive poorer service than others								
Never/Few times per year	150	33	1.00	Reference		1.00	Reference	
At least once per month	7	3	2.13	0.91	5.02	2.72	1.08	6.84
People act as if they are better than you								
Never/Few times per year	114	19	1.00	Reference		1.00	Reference	
At least once per month	43	17	2.91	1.68	5.07	2.90	1.68	5.00
People act as if you are dishonest								
Never/Few times per year	149	31	1.00	Reference		1.00	Reference	
At least once per month	8	5	3.24	1.72	6.09	3.87	1.98	7.54
People act as if they are afraid of you								
Never/Few times per year	150	32	1.00	Reference		1.00	Reference	
At least once per month	7	4	4.02	1.68	9.63	4.73	1.86	12.05
People act as if you are not intelligent								
Never/Few times per year	141	30	1.00	Reference		1.00	Reference	
At least once per month	16	6	1.95	0.92	4.12	2.33	1.07	5.08

^1^ Model 1 adjusted for age and history of poverty. ^2^ Model 2 adjusted for age, history of poverty, and alcohol consumption.

**Table 3:** Risk ratios and 95% confidence intervals for the association between individual measures of perceived lifetime discrimination and overweight/obesity (BMI ≥ 25 kg/m^2^), Epi AWARE Study (n = 157).

			Multivariable-Adjusted Relative Risks and 95% Confidence Intervals					
	N		Model 1 ^1^			Model 2 ^2^		
	Total	BMI ≥ 25 kg/m^2^	RR	95% CI		RR	95% CI	
*Have you been treated unfairly due to your race in any of the following circumstances?*								
Job (hiring, promotion, firing)								
No	112	22	1.00	Reference		1.00	Reference	
Yes	45	14	1.71	0.96	3.04	1.82	1.04	3.19
Housing (renting, buying, mortgage)								
No	147	35	1.00	Reference		1.00	Reference	
Yes	10	1	0.50	0.07	3.40	0.55	0.08	3.71
Police (stopped, searched, threatened)								
No	146	34	1.00	Reference		1.00	Reference	
Yes	11	2	0.83	0.22	3.24	0.94	0.25	3.50
In the courts								
No	156	36	1.00	Reference		1.00	Reference	
Yes	1	0	---	---	---	---	---	---
At School								
No	78	16	1.00	Reference		1.00	Reference	
Yes	79	20	1.69	0.89	3.21	1.69	0.89	3.20
Getting Medical Care								
No	142	28	1.00	Reference		1.00	Reference	
Yes	15	8	2.67	1.47	4.84	3.01	1.63	5.56

^1^ Adjusted for age and socioeconomic status (poverty). ^2^ Adjusted for age, socioeconomic status (poverty), and alcohol consumption.

**Table 4:** Relative risk ratios and 95% confidence intervals of cumulative perceived discrimination score and overweight/obesity (BMI 25 kg/m^2^ and 23 kg/m^2^) according to date of questionnaire completion (pre- or post-COVID-19 pandemic), Epi AWARE Study (n = 157).

	Questionnaire Completion on or Before 20 March 2020 (n = 78)				Questionnaire Completion After 20 March 2020 (n = 79)			
	N		Relative Risk (95% CI)		N		Relative Risk (95% CI)	
BMI Cut-Off: 25 kg/m^2^								
Cumulative Perceived Discrimination Score	Total	Overweight/Obese	Age-Adjusted	MV-Adjusted ^1^	Total	Overweight/Obese	Age-Adjusted	MV-Adjusted ^1^
No Reported Discrimination	26	6	1.00 (Reference)	1.00 (Reference)	28	3	1.00 (Reference)	1.00 (Reference)
Any Reported Discrimination	52	14	1.21 (0.53, 2.74)	1.93 (0.75, 5.01)	51	13	2.67 (0.81, 8.81)	2.65 (0.82, 8.58)
BMI Cut-Off: 23 kg/m^2^								
Cumulative Perceived Discrimination Score	Total	Overweight/Obese	Age-Adjusted	MV-Adjusted ^1^	Total	Overweight/Obese	Age-Adjusted	MV-Adjusted ^1^
No Reported Discrimination	26	11	1.00 (Reference)	1.00 (Reference)	28	7	1.00 (Reference)	1.00 (Reference)
Any Reported Discrimination	52	21	0.97 (0.56, 1.69)	1.06 (0.55, 1.83)	51	24	1.96 (0.98, 3.94)	1.92 (0.94, 3.90)

^1^ Adjusted for age and history of poverty.

## Data Availability

Data underlying the study cannot be made publicly available due to ethical concerns about patient confidentiality. Data will be made available to qualified researchers on request to epiaware@bu.edu.

## Supplementary Materials

The following supporting information can be downloaded at https://www.mdpi.com/article/10.3390/ijerph22050661/s1. Supplementary Table S1. Participant characteristics according to binary discrimination categories (no versus any reported discrimination), Epi AWARE Study (n = 157). Supplementary Table S2. Risk ratios and 95% confidence intervals for the association between individual measures of perceived everyday discrimination and overweight/obesity (BMI ≥ 23 kg/m^2^), Epi AWARE Study (n = 157). Supplementary Table S3. Risk ratios and 95% confidence intervals for the association between individual measures of perceived lifetime discrimination and body mass index (BMI ≥ 23 kg/m^2^), Epi AWARE Study (n = 157).
