# Supplementary material for: The Association Between Perceived Discrimination and Body Mass Index (BMI) Among Asian American Women Before and During the COVID-19 Pandemic
Source: Int J Environ Res Public Health. 2025 Apr 22;22(5):661. doi: 10.3390/ijerph22050661 (PMC12111472; doi:10.3390/ijerph22050661)
Supplement: Supplementary file 1 [file ijerph-22-00661-s001.zip › ijerph-3533668-supplementary.pdf]

International Journal of
Environmental Research and Public Health

MDPI

Supplemental Table S1. Participant Characteristics According to Binary Discrimination Categories (No versus Any Reported Discrimination), Epi AWARE Study (n = 157).

		Discrimination	
	**Total (N = 157)**	**No Reported Discrimination (N = 54)**	**Any Reported Discrimination (N = 103)**
**Age (years)**			
Mean (SD), Range	29.1 (9.4)<br>18.0, 59.0	30.3 (10.3)<br>19.0, 59.0	28.2 (8.8)<br>18.0, 57.0
		N (%)	
**Education (years)**			
> 12	152 (96.8%)	53 (98.2%)	99 (96.1%)
**Sexual Orientation**			
Heterosexual	123 (78.3%)	45 (83.3%)	78 (75.7%)
**Marital Status**			
Unmarried	122 (77.8%)	38 (70.4%)	84 (81.6)
**Moderate Physical Activity/Week**			
≥ 3 hours	30 (19.1%)	10 (18.5%)	20 (19.4%)
**Sedentary Activity/Day**			
≥ 5 hours	100 (63.7%)	34 (63.0%)	66 (64.1%)
**Country of Birth**			
United States	98 (62.4%)	33 (61.1%)	65 (63.1%)
**History of Poverty**			
Yes	60 (38.2%)	14 (25.9%)	46 (44.7%)
**Cigarette Smoking**			
Ever	16 (10.2%)	7 (13.0%)	9 (8.7%)
**Alcohol Consumption**			
Ever	24 (15.3%)	8 (14.8%)	16 (15.5%)
Missing	103 (65.6%)	34 (63.0%)	69 (67.0%)

Supplemental Table S2. Risk Ratios and 95% Confidence Intervals for the Association Between Individual Measures of Perceived Everyday Discrimination and Overweight/Obesity (BMI ≥ 23 kg/m^2), Epi AWARE Study (N = 157).

			Multivariable-adjusted Relative Risks and 95% Confidence Intervals					
	**N**		**Model 1** [1]			**Model** [2]		
	**Total**	**BMI ≥ 23 kg/m^2**	**RR**	**95% CI**		**RR**	**95% CI**	
In your day-to-day life, how often have any of the following things happened to you?								
**Receive poorer service than others**								
Never/Few time per year	150	60	1.00	Reference		1.00	Reference	
At least once per month	7	3	1.05	0.44	2.52	1.21	0.49	2.98

**People act as if they are better than you**								
Never/Few time per year	114	40	1.00	Reference		1.00	Reference	
At least once per month	43	23	1.57	1.07	2.30	1.53	1.05	2.25
**People act as if you are dishonest**								
Never/Few time per year	149	57	1.00	Reference		1.00	Reference	
At least once per month	8	6	1.95	1.21	3.12	2.11	1.31	3.43
**People act as if they are afraid of you**								
Never/Few time per year	150	58	1.00	Reference		1.00	Reference	
At least once per month	7	5	2.01	1.11	3.65	2.24	1.16	4.34
**People act as if you are not intelligent**								
Never/Few time per year	141	55	1.00	Reference		1.00	Reference	
At least once per month	16	8	1.28	0.74	2.20	1.36	0.79	2.34

[1] Adjusted for age, and socioeconomic status (poverty), [2] Adjusted for age, socioeconomic status (poverty) and alcohol consumption.

Supplemental Table S3. Risk Ratios and 95% Confidence Intervals for the Association Between Individual Measures of Perceived Lifetime Discrimination and Body Mass Index BMI ≥ 23 kg/m², Epi AWARE Study (N = 157).

			**Multivariable-adjusted Relative Risks and 95% Confidence Intervals**					
	**N**		**Model 1[1]**			**Model 2[2]**		
	**Total**	**BMI ≥ 23 kg/m²**	**RR**	**95% CI**		**RR**	**95% CI**	
Ever treated unfairly due to race in the following situations:								
**On the Job**								
No	112	48	1.00	Reference		1.00	Reference	
Yes	45	15	0.80	0.50	1.26	0.83	0.52	1.31
**Housing (renting, buying, mortgage)**								
No	147	62	1.00	Reference		1.00	Reference	
Yes	10	1	0.23	0.04	1.49	0.24	0.04	1.56
**Police (stopped, searched, threatened)**								
No	146	61	1.00	Reference		1.00	Reference	
Yes	11	2	0.42	0.12	1.51	0.44	0.13	1.56
**In the courts**								
No	156	93	1.00	Reference		1.00	Reference	
Yes	1	0	---	---	---	---	---	---
**At School**								
No	78	29	1.00	Reference		1.00	Reference	
Yes	79	34	1.32	0.87	1.98	1.28	0.85	1.92
**Receiving Medical Care**								
No	15	11	1.00	Reference		1.00	Reference	
Yes	142	52	1.95	1.33	2.86	2.12	1.46	3.08

[1] Adjusted for age, and socioeconomic status (poverty). [2] Adjusted for age, socioeconomic status (poverty) and alcohol consumption.
